# Efficacy and tolerability of mono-compound topical treatments for reduction of intraocular pressure in patients with primary open angle glaucoma or ocular hypertension: an overview of reviews

**DOI:** 10.3325/cmj.2014.55.468

**Published:** 2014-10

**Authors:** Qëndresë Daka, Vladimir Trkulja

**Affiliations:** 1 Department of Ophthalmology, University Clinical Centre of Kosovo, Medical School, Prishtina University, Prishtina, Kosovo; 2 Department of Pharmacology, University of Zagreb School of Medicine, Zagreb, Croatia

## Abstract

**Aim:**

To evaluate the existing evidence on relative efficacy and tolerability of topical mono-compound intraocular pressure (IOP)-lowering drugs in treatment of primary open angle glaucoma (POAG) and ocular hypertension (OHT).

**Methods:**

In this systematic review of systematic reviews/meta-analyses of randomized controlled trials a thorough and sensitive search of PubMed, Embase and Cochrane Databases was performed. Individual study methodological quality and quality of evidence were assessed using the AMSTAR checklist and the GRADE system, respectively. The relationships between individual drugs were evaluated based on the best available evidence.

**Results:**

Of the 133 initial non-duplicate records, 16 studies met the inclusion criteria. Five achieved an overall “moderate” (none achieved “high”) quality of evidence and evaluated prostaglandin analogues (PGAs) – latanoprost, travoprost, and bimatoprost; timolol; “other beta-blockers;” carbonic anhydrase inhibitors (CAI) as a group or dorzolamide separately; and brimonidine. “Moderate quality” refers to efficacy and incidence of conjunctival hyperemia. Quality of evidence regarding other tolerability aspects was low. PGAs should be considered equivalent regarding efficacy, but latanoprost was relevantly better tolerated than the other two. Non-PGA compounds did not relevantly differ between each other in either efficacy or safety. Timolol and brimonidine were relevantly less effective than all PGAs. The same was true for CAI vs bimatoprost. Regarding tolerability, timolol was superior to all PGAs and brimonidine and CAI were superior to bimatoprost.

**Conclusion:**

No high quality evidence on relative efficacy and tolerability of the most commonly used mono-compound IOP-lowering drugs for POAG/OHT exists. Moderate quality evidence indicates latanoprost as a treatment with the most favorable trade-off between benefits and harms.

Glaucoma subsumes a group of optic neuropathies with different causes and pathophysiological mechanisms that can permanently damage vision in the affected eye if left untreated (1,2). It is the leading cause of irreversible blindness worldwide (2-5). Depending on the mechanism of aqueous outflow impairment with respect to the anterior chamber angle configuration, glaucoma is classified as open or closed angle glaucoma. Open angle glaucoma (OAG) is by far more common (75%) and it is estimated that by the year 2020 it will affect 60 million people worldwide (2,4). Depending on the presence or absence of ocular or systemic disorders, OAG is classified as primary or secondary, with the primary form (POAG) prevailing. POAG is most commonly characterized by increased intraocular pressure (IOP), but IOP could also be consistently “normal”, ie, within ±2-3 standard deviations of an average “normal” value (6). Consequently, it is denoted as “high-tension” POAG (POAG-HTG) or as “normal-tension” POAG (POAG-NTG). In any case, it is a progressive chronic optic neuropathy in the absence of identifiable causes, where IOP and other unknown factors contribute to the loss of retinal ganglionic cells (RGCs) and their axons (7-9). When IOP is elevated but there is no detectable glaucomatous damage, the condition is called ocular hypertension (OHT) (8). Damage to the optical nerve is usually slow, and if treated, most patients retain useful vision for their entire lives. Therapeutic aim is to prevent impairment of vision by slowing down the apoptosis of RGCs. Different treatment modalities targeting factors that may play a role in POAG pathogenesis are being investigated (10-12) but for the time being, the only modality recommended by the professional guidelines is IOP-lowering treatment (7,8). Reduction of IOP prevents both conversion of OHT to POAG and progression of POAG (13-16), and can be achieved by medications, laser, or surgical therapy. Topical drug therapy is a standard initial intervention, whereas the latter two options are implemented mainly when conservative therapy is not effective, not tolerated, or not utilized by the patient (7-9). There are five major classes of IOP-lowering compounds, each comprising several individual drugs, and also a large number of their fixed combinations. They lower IOP by reducing aqueous production or/and by increasing aqueous outflow (7,17-20). According to the guidelines, IOP-lowering treatment should start with a mono-compound therapy and should aim to reduce IOP by 20%-30%. A rational first line mono-compound drug is the one installed as infrequently as possible for the therapeutic effect and with the fewest side-effects (7,8). If the first line medication is not effective or not tolerated it could be substituted, another drug may be added (unfixed combination), or a patient could be switched to a fixed combination of different compounds (8,17,18). A recent comprehensive evaluation (9,21) demonstrated a high level of evidence of efficacy in IOP reduction of various topical pharmacological treatments but with some uncertainty regarding their mutual relationship in this respect. The latter was in part due to inconsistent results of some studies and to the complexity of the setting (eg, different forms of OAG, treatment-naďve/previously unsuccessfully treated patients) and treatment modalities (mono-compounds, fixed/unfixed combinations) (9,21). We aimed to evaluate the existing evidence on relative efficacy and tolerability of mono-compound topical medications (recommended as initial treatment options) in POAG and OHT, the most common conditions requiring IOP-reducing therapy. Since by the year 2010 there were already 112 systematic reviews published on various aspects of glaucoma (22), of which at least 20 addressed medical treatments (23), we decided to perform an overview of systematic reviews, as they are generally considered a (potential) source of the highest level of evidence about therapeutic interventions.

## Materials and methods

We performed an overview of systematic reviews/meta-analyses of randomized controlled trials (RCTs) of IOP-lowering mono-compound treatments in patients with POAG and/or OHT. The PRIMSA statement (24) and the current Cochrane Handbook for Systematic Reviews (Version 5.1.0, 2011) (25) were consulted for evaluation of reporting and technical aspects of the included reviews. The overview of reviews followed the format suggested by the Cochrane Handbook (25).

### Search strategy

The initial search was conceived to be sensitive, not specific, hence no restrictions were set. We searched electronic databases – PubMed, Embase, and all Cochrane Databases (Cochrane Database of Systematic Reviews, Health Technology Assessment Database, Database of Abstracts of Reviews of Effects) – till January 31, 2014 and performed a further search of reference lists of relevant publications. In PubMed, “clinical queries” and “related articles” functions were used to broaden the search. Direct search terms and controlled terms were adjusted to each database. To identify study design, we used “systematic review,” “meta-analysis,” “RCT;” to identify the health condition, we used “glaucoma,” “open angle glaucoma,” “ocular hypertension;” and to search for medications, we used “therapy,” “treatment,” “intervention,” “drug,” and all individual names of known IOP-lowering compounds. The common search strategy was implemented by the two investigators independently.

### Selection of reviews

The reviews were included when they met the following criteria: a) they had to be systematic reviews with/without meta-analysis; b) they had to include only primary trials in POAG/OHT patients (ie, at least 85% POAG/OHT patients in the trial), or it could be verified that at least 85% of the patients across the included primary trials were POAG/OHT patients; c) they had to evaluate the efficacy and/or safety of mono-compound topical IOP-lowering medications. Systematic reviews/meta-analyses focused exclusively on combinational treatment modalities (unfixed or fixed combinations) were not included. After removal of duplicate publications, studies were screened based on titles and abstract to select those for full text assessment of eligibility. Reviews were selected independently by the two investigators and disagreements were resolved by a consensus.

### Data abstracting

Two investigators independently abstracted the selected reviews. Collection forms were compared and disagreements resolved by a consensus. The following characteristics were recorded: search strategy particulars, study selection criteria, declared purpose of the review, number of included trials and patients, evaluated treatments, pair-wise comparisons, assessed outcomes, quality assessment of primary trials, methods for data pooling, reporting on heterogeneity, exploration of heterogeneity, evaluation of publication bias, presentation of primary study data, sensitivity analyses, and funding source. Meta-analysis results were also extracted and assessed for consistency and precision.

### Assessment of methodological quality of reviews

Methodological quality of the included reviews was assessed independently by the two investigators using the AMSTAR checklist (26). This validated tool assesses 11 items formulated as questions pertaining to quality (26) and each may be answered as “Yes,” “No,” “Can’t answer (?),” and “Not applicable.” Only a “Yes” assigns a point to an item. Maximum possible score is 11. Inter-rater agreement of the checklist is good (26). In the present overview, discrepancies between the two checklists (investigators) were found only in 15 (8.5%) items out of 176 assessed by each investigator (16 systematic reviews ×11 items) and were resolved by a consensus.

### Synthesizing data as quality of evidence

We used the GRADE system (27) to derive the level of evidence provided by each systematic review and then to derive the level of evidence (based on all reviews) regarding individual mono-compound relationships regarding IOP-lowering efficacy and safety. For systematic reviews/meta-analyses, the GRADE system assigns an *a priori* high level of “quality of evidence,” and then addresses potential drawbacks (“-1 step” if serious, “-2 steps” if very serious) regarding bias, inconsistency, indirectness, imprecision, and publication bias; ie, potential additional strengths (“+1 step” or “+2 steps”) regarding the size of the effect, evidence of dose-response, and accounting for residual confounding (27). The final grades of quality of evidence are “high” (strong confidence that the true effect lies close to the estimate of the effect); “moderate” (moderate confidence in the estimate: the true effect is likely to be close, but there is a possibility that it is substantially different); “low” (limited confidence about the estimate: the true effect may substantially different) and “very low” (little confidence in the effect estimate: the true effect is likely to be substantially different).

## Results

### Selection of reviews

**Figure 1:**
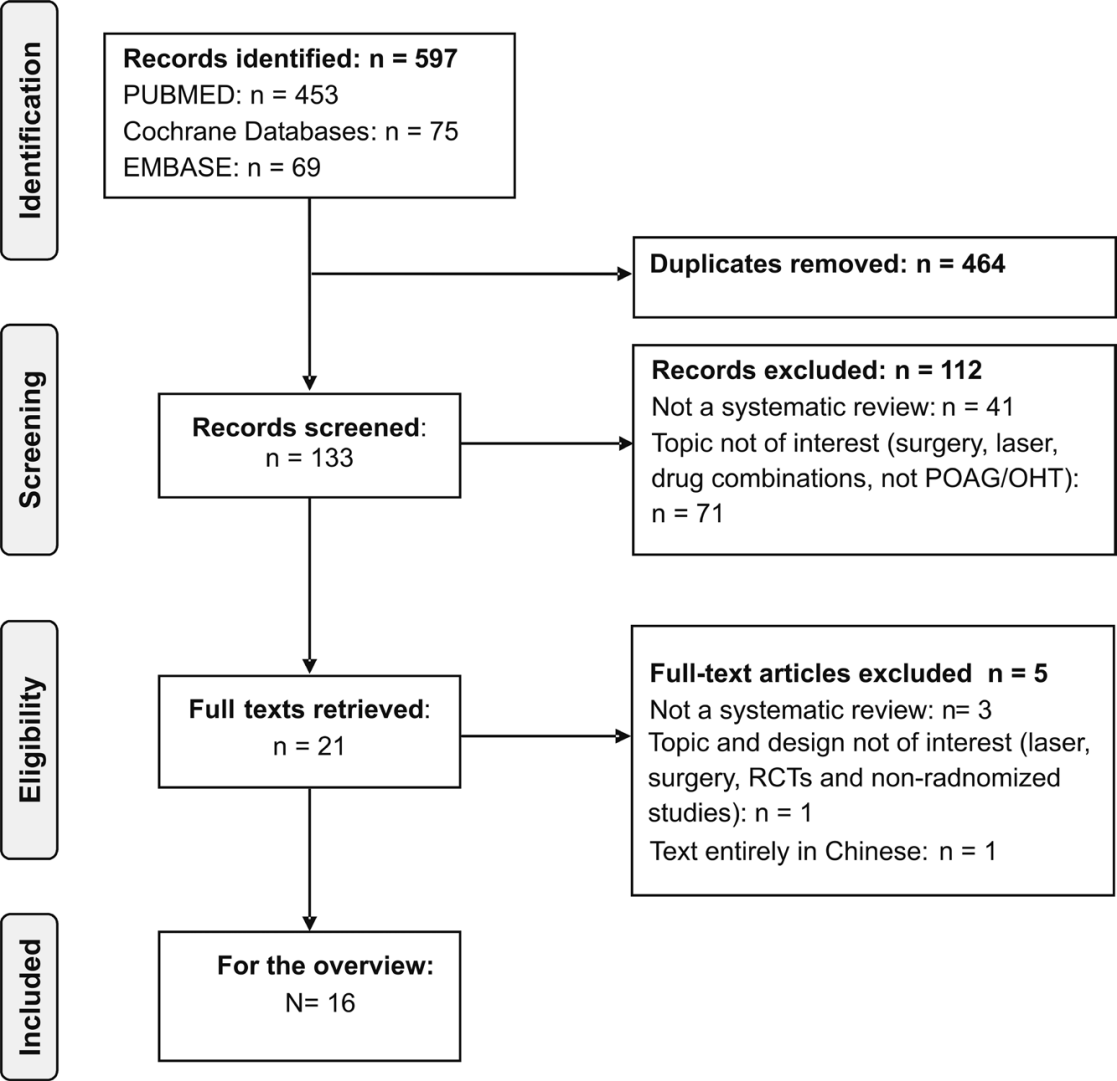
PRISMA flow-chart of the study selection process.

Of the initially identified 597 records (none by the hand search), 133 non-duplicates were screened and 21 retrieved in full text. Finally, the present overview included 16 systematic reviews (Figure 1) (see Supplementary Table 1 (web extra material 1) for those excluded in the last step).

### Characteristics of the included reviews

Table 1 summarizes main characteristics of the 16 included reviews. They were published between 2000 and 2010. Most (10/16) were focused on both efficacy and tolerability/safety (as a secondary objective) (29,31,33-38,40,43), five addressed only efficacy (28,30,32,39,42), and one addressed exclusively local tolerability, specifically conjunctival hyperemia (41). Two reviews (42,43) synthesized data using network meta-analysis, whereas others were declared as “classical” meta-analyses. Two authors published more than one review [Cheng et al (35,38,39)], even on the same primary studies [Van der Valk et al (30,42)]. The assessed treatments and primary study selection criteria varied. One network meta-analysis (42) aimed to assess “the most commonly prescribed mono-compounds,” whereas the other (43) evaluated also combination treatments, but the focus of the present overview was on mono-compounds. Another review (33) also addressed mono-compound and “adjunctive treatment” comparisons, but was included as it provided the most comprehensive comparison between latanoprost and brimonidine. Two further reviews evaluated “all most commonly prescribed mono-compound treatments” (30,39). Overall, the following mono-compound medications were evaluated through different modes of mutual comparisons: placebo; beta-blockers (BB) – timolol (always as an individual compound), betaxolol (as an individual compound or referred to as “other BB” together with eg, carteolol, levobunolol, and others); alpha2-receptor agonists – brimonidine; carbonic anhydrase inhibitors (CAI) – brinzolamide and dorzolamide (as individual compounds or as “CAI as a group”); and prostaglandin analogues (PGAs) – latanoprost, travoprost, and bimatoprost. One review (39) explicitly included only trials in normal-tension glaucoma (NTG) patients. Considering the inclusion/exclusion criteria for primary studies combined with the displayed structures of the embraced patients (in the primary studies), it is safe to conclude that all other reviews assessed the treatments in the setting of (predominantly) POAG/OHT. Only 1/16 reviews included, among the primary studies, several non-randomized trials (4/16) (40). All other primary trials in all other reviews were RCTs, although 2 reviews intended to include also quasi-randomized trials (33,37). Two reviews (32,43) did not assess primary study quality and the others implemented different tools, most commonly the Jadad score.

**Table 1:** Main characteristics of the included reviews (in chronological order)*^†^

Reference	Objective	Criteria for primary studies	Studies (k), patients (n)	Primary study assessments
Einarson 2000 (28)	Indirectly compare LAT with BRIM for IOP reduction in POAG.	RCT, English language. POAG with IOP≥20 mm Hg. At least one arm includes LAT or BRIM. Peak, trough or diurnal IOP; duration 3-12 months.	k = 9 (DB, parallel), none comparing LAT to BRIM. LAT: k = 6; BRIM: k = 3.	Quality: Jadad score. Efficacy: ΔIOP vs baseline and % with controlled IOP for LAT and BRIM as individual treatments. Safety: not assessed. Random-effects.
Zhang 2001 (29)	Compare LAT with TIM for IOP reduction and safety in OAG/OHT.	RCT. OAG/OHT. Directly compare LAT and TIM.	k = 11 (10 DB, 1 SB; 7 parallel, 4 crossover); n = 1256; 410 POAG, 465 OHT, 137 OAG.	Quality: Jadad score. Efficacy: ΔIOP vs baseline at 4 time points. Safety: local, systemic, AE withdrawals. Random or fixed-effect.
van der Valk 2005 (30)	Estimate IOP reduction at peak and trough by the most commonly prescribed mono compounds in POAG-HTG/OHT.	RCT, English, German, Dutch or French language. POAG-HTG or OHT. Compare (any): Placebo; TIM 0.5% bid; BET 0.5% bid; BRIM 0.2% bid; DORZ 2.0% bid; BRINZ 1.0% tid; LAT 0.005% qd; TRAV 0.004% qd; BIMA 0.03% qd.	k = 27, n = 6053 for peak and 6861 for trough IOP. Placebo k = 3; BET k = 5; TIM k = 15; BIMA k = 6; LAT k = 12; TRAV k = 5; BRIM k = 4, BRINZ k = 1, DORZ k = 6.	Quality: Delphi score. Efficacy: absolute and relative ΔIOP vs baseline for peak and trough for each individual treatment; 1-6 months pooled as 1 time point. Safety: not assessed. Random-effects.
Li 2006 (31)	Compare TRAV with LAT, BIMA and TIM for IOP reduction and safety in OAG/OHT.	RCT, English or Chinese language. OAG or OHT; Compare TRAV vs other PGA or TIM. Report IOP or AEs.	k = 12 (parallel, 8 DB, 4 SB); n = 3048, 2060 POAG, 840 OHT, 114 other. TRAV 0.004% vs TIM k = 4; TRAV 0.004% vs BIMA k = 5; TRAV 0.004% vs LAT k = 5.	Quality: Cochrane tool for risk of bias. Efficacy: ΔIOP vs baseline; 8/12 trials ITT analysis. Safety: local. Random or fixed-effect.
Denis 2007 (32)	Compare TRAV with LAT and BIMA for IOP reduction in OAG/OHT.	RCT, parallel, English of French language. OAG or OHT. Any comparison of TRAV, LAT, BIMA; Report on IOP.	k = 9; n = 1318, 378 OHT, 919 OAG, 21 other Comparing all 3 (three-arm trials) k = 2 Comparing any two (two-arm trials) k = 7.	Quality: not assessed. Efficacy: IOP at study end (average) and % responding for each individual treatment. Safety: Not assessed. Random-effects.
Fung 2007 (33)	Compare LAT with BRIM for IOP reduction and safety in OAG/OHT.	RCT or quasi-RCT. OAG/NTG/OHT. Compare LAT to BRIM; adjunctive treatment possible. Duration ≥1 month. Reports on efficacy or safety.	k = 15 (all RCT, 11 parallel, 4 crossover; 4 DB, 7 SB, 4 unknown). n = 1824, 1299 OAG, 390 OHT, 64 NTG, 60 other. Mono-treatment k = 9; adjunctive treatment k = 6.	Quality: Allocation concealment, blinding and IOP measurement method. Efficacy: ΔIOP vs baseline, peak or diurnal. 5/15 trials ITT analysis. Safety: local and systemic. Random-effects.
Aptel 2008 (34)	Compare BIMA, LAT and TRAV for IOP reduction and safety in POAG/OHT.	RCT, DB. POAG or OHT>90%. Compare LAT 0.005%, TRAV 0.004% or BIMA 0.03% 1 drop/d between 6 and 10 pm Report diurnal IOP and conjunctival hyperemia. Duration 1-6 months.	k = 8 (parallel). n = 1610; LAT vs BIMA k = 4; TRAV vs BIMA k = 2; LAT vs TRAV k = 1; LAT vs TRAV vs BIMA k = 1.	Quality: Jadad score. Efficacy: Δ IOP vs baseline (4 daily values: 8 am, 12 noon, 4 pm, 8 pm separately), all trial duration as one time point. Safety: conjunctival hyperemia. Fixed-effect.
Cheng 2008 (35)	Compare BIMA with LAT for IOP reduction and safety in glaucoma/OHT.	RCT. Glaucoma or OHT, NTG excluded. Directly compare LAT and BIMA. Report on IOP reduction or % patients achieving the target IOP.	k = 13 (5 DB, 8 SB, 10 parallel, 3 crossover). n = 1302; 754 POAG, 327 OHT, 211 other. LAT 0.005% vs BIMA 0.03%; 1 × evening	Quality: Jadad score. Efficacy: Δ IOP vs baseline (morning/diurnal) or % patients achieving IOP≤17; 3 different time points. Safety. local. Random-effects. ITT basis.
Hodge 2008 (36)	Compare PGAs with BRIM and DORZ for IOP reduction and safety in OAG/OHT.	RCT, English language. OAG/OHT, ACG excluded. Compare PGAs and BRIM or DORZ.	k = 7 (parallel); n = 1131, 418 POAG, 555 OHT, 60 other, 98 unknown. LAT vs BRIM k = 3 (+1 safety); LAT vs DORZ k = 3	Quality: Jadad score. Efficacy: Δ IOP vs baseline at 3 months. 2/7 trials ITT. Safety: local, AE withdrawals. Random or fixed-effect.
Loon 2008 (37)	Compare TIM with BRIM for IOP reduction and safety in glaucoma.	RCT, pseudo-RCT. Glaucoma. Directly compare TIM 0.5% to BRIM 0.2%. Report on IOP and safety, ≥1 month.	k = 10 (all RCT, 7 DB, 1 open, 2 unknown); 8 used for quantitative synthesis; n = 2387, 1442 OAG, 877 OHT, 68 other.	Quality: Allocation concealment, blinding, withdrawals, ITT/LOCF. Efficacy: Δ IOP vs baseline (peak or mean). 8/10 trials ITT analysis. Safety: local, systemic. Random-effects.
Cheng 2009a (38)	Compare TRAV and LAT for IOP reduction and safety in OAG/OHT.	RCT. OAG/OHT with lOP>21 mm Hg, NTG/ACG excluded. Compare TRAV 0.004% to LAT 0.005% once daily. Report on IOP at 9 am and/or 5 pm	k = 17 (9 DB, 8 SB, 13 parallel, 4 crossover), n = 1491; 966 OAG, 379 OHT, 146 other.	Quality: Jadad score. Efficacy: Δ IOP vs baseline (separately 9 am and 5 pm) at 5 different time points. 6/17 trials ITT analysis. Safety: local and AE withdrawals. Random-effects.
Cheng 2009b (39)	Estimate IOP reduction by the most commonly prescribed mono compounds in NTG.	RCT, any language; Advanced NPG. Compare (any): Placebo, BET 0.25/0.5% bid, TIM 0.5% bid, DORZ 0.2% tid, BRINZ 1.0% tid, BRIM 0.2% bid, LAT 0.005% qd, TRAV 0.004% qd, BIMA 0.03% qd. Report absolute and relative IOP reduction.	k = 15 (5 DB, 6 SB, 4 open, 5 parallel, 10 crossover); n = 450.	Quality: Delphi score. Efficacy: absolute and relative ΔIOP vs baseline for peak, trough and diurnal curve for each individual treatment (0.5-2 months) as 1 time point. Safety: not assessed. Random-effects.
Ejawo 2009 (40)	Compare BIMA, LAT and TRAV for IOP reduction and safety in POAG/OHT.	RCT, excluded dose-finding, crossover and short-term. POAG/OHT. Any comparison between TRAV 0.004%, LAT 0.005% and BIMA 0.03%. Report on IOP and AEs.	k = 16 (4 non-RCT, unknown blinding), n = 2674, 1705 POAG, 727 OHT, 242 other. TRAV vs LAT k = 9; TRAV vs BIMA k = 8; LAT vs BIMA k = 8; >2 arms k = 5.	Quality: randomization, allocation concealment, ITT, blinding. Efficacy: IOP (morning) at study end (3-12 months), 6/16 trials ITT analysis. Safety: conjunctival hyperemia. Random-effects.
Hornubia 2009 (41)	Compare LAT with BIMA and TRAV for conjunctival hyperemia in glaucoma/OHT.	RCT, English language. Glaucoma/OHT. Any comparison between LAT, BIMA or TRAV reporting on conjunctival hyperemia.	k = 13 (10 parallel, 3 crossover); n = 2222; 1364 OAG, 678 OHT and 180 other. LAT vs BIMA k = 8, LAT vs TRAV k = 6, 3 arms k = 1.	Quality: Jadad score. Efficacy: Not assessed. Safety: Conjunctival hyperemia. Fixed and random-effects.
v.d.Valk 2009 (42)	Estimate IOP reduction at peak and trough by the most commonly prescribed mono compounds in POAG-HTG/OHT by MTC.	RCT, English, German, Dutch or French language. POAG-HTG or OHT. Compare (any): Placebo; TIM 0.5% bid; BET 0.5% bid; BRIM 0.2% bid; DORZ 2.0% bid; BRINZ 1.0% tid; LAT 0.005% qd; TRAV 0.004% qd; BIMA 0.03% qd.	k = 27, n = 6053 for peak and 6861 for trough IOP. Placebo k = 3; BET k = 5; TIM k = 15; BIMA k = 6; LAT k = 12; TRAV k = 5; BRIM k = 4, BRINZ k = 1, DORZ k = 6.	Quality: Delphi score. Efficacy: absolute and relative ΔIOP vs baseline for peak and trough using timolol as a reference;1-6 months pooled as 1 time point. Safety: not assessed. Random-effects.
Orme 2010 (43)	Compare IOP reduction and conjunctival hyperemia of different treatments in POAG/OHT by MTC.	RCT, English language, ≥20 patients. POAG/OHT, excluded ACG & secondary. Include a PGA in at least one arm.	MTC Efficacy: k = 18; n = 2943; MetaReg Efficacy k = 73, n = 11519; MTC Safety: k = 72. Evaluated treatments: TIM, LAT- TlM, CAI-TIM, TRAV-TIM, BIMA, TRAV, LAT, CAI, Other UC, Other BB, Placebo.	Quality: Not assessed. Efficacy: MTC for 3-month outcomes - absolute IOP; predicted probability of IOP<20 mm Hg or ≥20% reduction vs baseline and NNTB vs timolol. Safety: MTC of % patients with conjunctival hyperemia and NNTH vs Placebo. Random-effects.

*All individual studies included men and women, but their proportions varied.

†Abbreviations related to methodology: RCT – randomized controlled trial; DB – double-blind; SB – single blind; ITT – intention–to-treat; LOCF – last observation carried-forward; PP – per-protocol; NNTB – number needed to treat to benefit; NNTH – number needed to treat to harm; MetaReg – meta-regression analysis; MTC – random-effects mixed treatment comparisons (or network meta-analysis);

Abbreviations related to disease: POAG – primary open angle glaucoma; OHT – ocular hypertension; NTG – normal tension glaucoma; OAG – open angle glaucoma; PDG – pigment dispersion glaucoma; ACG – angle closed glaucoma; HTG – high tension glaucoma; IOP – intraocular pressure; AEs – adverse events;

Abbreviations related to drugs: PGAs – prostaglandin analogues; CAI – carbonic anhydrase inhibitors; BB – beta-blockers; UC – unfixed combinations; LAT – latanoprost; BIMA – bimatoprost; TRAV – travoprost; BRIM – brimonidine; TIM – timolol; BET – betaxolol; DORZ – dorzolamide; BRINZ – brinzolamide; qd – once a day; bid – twice a day; tid – three times a day.

### Quality of the included reviews

The AMSTAR checklist for the included reviews is shown in Table 2. Rationale for the assigned scores is elaborated in more detail in Supplementary Table 2(web extra material 2). Some elements of scoring are self-evident (eg, whether conflict of interest was declared or publication bias assessed), but some require clarifications, particularly if “N” or “?” was assigned to an item. We assigned “?” for the first AMSTAR item to four studies due to discrepancies between the declared aims and actually implemented procedures. For example, two reviews (30,39) intended to evaluate a large number of individual treatments without defining the method, but did not use network meta-analysis (for details see Supplementary Table 2(web extra material 2)). The reason for assigning “?” to the fifth AMSTAR item in most of the reviews was the fact that the lists of the included but not the lists of excluded primary trials were reported. Reviews that did not consider primary trial quality when drawing conclusions from the meta-analytical results were assigned an “N” for the eighth AMSTAR item. However, the major flaws were related to methods of data pooling (see Supplementary Table 2(web extra material 2) for details): only 4 reviews used fully correct methods; 5 reviews were assigned a “?” as they alternately (and inappropriately) used fixed and random-effects pooling without clear criteria; and 7 reviews used explicitly erroneous data pooling methods that resulted in non-randomized comparison and/or erroneous variance calculation.

**Table 2:** Quality of the included reviews based on the AMSTAR (26) checklist*

	Einarson 2000 (28)	Zhang 2001 (29)	v.d Valk 2005 (30)	Li 2006 (31)	Denis 2007 (32)	Fung 2007 (33)	Aptel 2008 (34)	Cheng 2008 (35)	Hodge 2008 (36)	Loon 2008 (37)	Cheng 2009a (38)	Cheng 2009b (39)	Ejawo 2009 (40)	Hornubia 2009 (41)	v.d.Valk 2009 (42)	Orme 2010 (43)
Design “*a priori*”?	?	Y	?	Y	Y	Y	Y	Y	Y	Y	Y	?	?	Y	Y	Y
Duplicate selection/extraction?	Y	Y	Y	Y	N	Y	Y	Y	Y	Y	Y	Y	Y	Y	Y^‡^	?
Comprehensive search?	Y	Y	Y	Y	Y	Y	Y	Y	Y	Y	Y	Y	Y	Y	Y^‡^	Y
Publication status clear?	Y	Y	Y	Y	Y	Y	Y	Y	Y	Y	Y	Y	Y	Y	Y^‡^	Y
List included/excluded provided?^†^	?	?	?	Y	?	?	?	Y	?	?	?	?	?	Y	?	Y
Study characteristics provided?	Y	Y	Y	Y	Y	Y	Y	Y	Y	Y	Y	Y	Y	Y	Y^‡^	Y
Quality assessed?	Y	Y	Y	Y	N	Y	Y	Y	Y	Y	Y	Y	Y	Y	Y^‡^	N
Quality accounted for conclusions?	Y	Y	Y	N	N	Y	Y	N	Y	Y	Y	Y	Y	N	N	N
Appropriate method for pooling?	N	N	N	?	N	Y	?	?	Y	N	N	N	Y	?	?	Y
Publication bias assessed?	N	N	Y	Y	Y	Y	Y	N	N	Y	Y	Y	Y	Y	?	Y
Conflict of interest declared?	Y	N	Y	Y	Y	Y	Y	Y	Y	N	N	Y	Y	Y	Y	Y
AMSTAR score	7	7	8	9	6	10	9	8	9	8	8	8	9	9	7	8

*Abbreviations: Y – yes; N – No; ? – can’t tell; NA – not applicable.

†All reviews reported on included studies, but only 3 reported also on excluded studies. Hence, most reviews failed to meet this quality criterion.

‡Described in the previous publication [v.d. Valk 2005 (30)].

### Quality of evidence

The highest level of quality of evidence achieved was “moderate” and was attained by 5 reviews (Table 3). Li et al, 2006 (31) and Aptel et al, 2008 (34) were downgraded by 1 for imprecision because certain comparisons between pairs of treatments were based on only 1-2 primary trials and/or a small number of patients, resulting in very wide confidence intervals. Fung et al, 2007 (33) and Hornubia et al, 2009 (41) were downgraded by 1 for limitations/bias since they included only a few double-blind trials and intent-to-treat analysis in primary studies was low or unknown. In addition, the unit-of-analysis issue (unclear handling of multi-arm and crossover trials) was highly suspected in Hornubia et al, 2009 (41). Orme et al, 2010 (43) was downgraded by 1 for indirectness since some of the efficacy comparisons in the assessed network were predominantly or exclusively indirect. Details on rationale for quality assessment of these and all other reviews are available in Supplementary Table 2(web extra material 2).

**Table 3:** Quality of evidence provided by individual reviews based on the GRADE (27) evaluation system

	Einarson 2000 (28)	Zhang 2001 (29)	v.d Valk 2005 (30)	Li 2006 (31)	Denis 2007 (32)	Fung 2007 (33)	Aptel 2008 (34)	Cheng 2008 (35)	Hodge 2008 (36)	Loon 2008 (37)	Cheng 2009a (38)	Cheng 2009b (39)	Ejawo 2009 (40)	Hornubia 2009 (41)	v.d.Valk 2009 (42)	Orme 2010 (43)
Limitations/bias	-1	-1	-1	Minor	-2	-1	Minor	-1	-1	-1	-1	-1	-2	-1	-1	Possible
Inconsistency	Minor	-1	Minor	Minor	-1	Minor	Minor	-1	Some	-1	Minor	Minor	Some	No	Minor	No
Indirectness	-2	Direct	-2	Direct	-2	Direct	Direct	Direct	Direct	Direct	Direct	-2	Direct	Direct	-1	-1
Imprecision	Minor	Minor	Minor	-1	Minor	Minor	-1	Some	-1	Minor	-1	Some	Minor	Minor	Minor	Minor
Publication bias	Unlikely	Unlikely	Unlikely	Unlikely	Unlikely	Unlikely	Unlikely	Unlikely	Unlikely	Unlikely	Unlikely	Unlikely	Unlikely	Unlikely	Possible	Unlikely
Quality of body of evidence*	+ Very low	++ Low	+ Very low	+++ Moderate	- Very low	+++ Moderate	+++ Moderate	++ Low	++ Low	++ Low	++ Low	+ Very low	++ Low	+++ Moderate	++ Low	+++ Moderate

*This is judged in respect to the primary research question posted in each review. See Materials and Methods for the GRADE system levels of quality of evidence.

### Relationship between different compounds

Only the reviews achieving moderate level of quality of evidence (31,33,34,41,43) were considered for evaluation of relationship between treatments.

*Efficacy.* Based on IOP reduction after 3 months of treatment, Orme et al (43) ranked 6 mono-compound medications in the following order (most effective to least effective): bimatoprost, latanoprost, travoprost, CAI group, BB group without timolol, timolol. However, many of the differences between treatments were very small and although statistically significant they did not appear practically relevant. Table 4 summarizes point-estimates of pair-wise differences in IOP reduction reported by Orme et al (43). Assuming that the limits of -1.0 to +1.0 mm Hg for IOP reduction are reasonable limits of therapeutic equivalence (40), the three PGAs appear to be equivalent. In a series of pair-wise comparisons, Aptel et al (34) calculated somewhat larger differences between bimatoprost and latanoprost, and between bimatoprost and travoprost – but still, all were well within the -1.0 to +1.0 range. Li et al (41) compared travoprost to bimatoprost and reported zero difference (point-estimate 0.08 mm Hg), while the difference between travoprost and latanoprost was -0.57 mm Hg. Despite these variations, it appears reasonable to conclude that the IOP-reducing potential of the three PGAs is not relevantly different.

**Table 4:** Point-estimates of differences (mmHg) between pairs of treatments in IOP reduction at 3 months vs baseline as determined in a network meta-analysis by Orme et al (43). A positive value indicates a greater reduction by the “row drug” vs “column drug” and a negative value indicates the opposite. Bolded are values that exceed a difference of ±1.0 mm Hg and underlined are the differences close to this limit*^†^

	Bimatoprost	Latanoprost	Travoprost	CAI as a group	BB w/o timolol	Timolol
Bimatoprost	—	0.45	0.47	0.97	**1.09**	**1.41**
Latanoprost	-0.45	—	0.02	0.52	0.64	0.96
Travoprost	-0.49	-0.02	—	0.50	0.62	0.94
CAI as a group	-0.97	-0.52	-0.50	—	0.12	0.44
BB w/o timolol	**-1.09**	-0.64	-0.62	-0.12	—	0.32
Timolol	**-1.41**	-0.96	-0.94	-0.44	-0.32	—

*We assumed that the limits of -1.0 to +1.0 mm Hg for a difference in IOP reduction could be reasonably accepted as limits of equivalence (40).

†IOP – intraocular pressure; BB – beta blockers; CAI – carbonic anhydrase inhibitors; w/o – without.

According to Orme et al (43), PGAs are more effective than other evaluated drugs, but in this respect they should be considered individually. Bimatoprost was relevantly superior to CAI, BB other than timolol, and timolol, whereas latanoprost and travoprost showed a relevant difference only in relation to timolol (Table 4). The findings of Li et al (41) confirmed the size of the difference between travoprost and timolol, whereas Fung et al (33) reported a relevant difference between latanoprost and brimonidine (point-estimate 1.10 mm Hg). Finally, according to Orme et al (43), CAI as a group, timolol, and other BB did not relevantly differ regarding their IOP-reducing potential (Table 4). However, these relationships were estimated practically exclusively through indirect comparisons and we found no other evidence of at least moderate quality that would relate these treatments to each other.

*Tolerability/safety.* The only adverse event addressed by all 5 reviews was conjunctival hyperemia. The most comprehensive assessment was that by Orme et al (43), ie, through a network meta-analysis including 73 RCTs. Ranking of mono-compound drugs from the lowest to the highest incidence of conjunctival hyperemia was: timolol, dorzolamide, brimonidine, latanoprost, travoprost, and bimatoprost. Betaxolol was also ranked, but based only on one trial arm with only 34 patients (43). Table 5 summarizes point-estimate odds ratios (ORs). Odds of hyperemia with latanoprost were around 3-fold and around 5-fold lower than with travoprost and bimatoprost, respectively (Table 5). Similar estimates were reported by Hornubia et al (41), whereas estimates provided by Aptel et al (34) and Li et al (41) were somewhat smaller (1.5-2.0-fold lower odds). While Orme et al (43) indicated no relevant difference between travoprost and bimatoprost (Table 5), Aptel et al (34) and Li et al (41) reported a significantly lower incidence with travoprost – ORs around 0.86 (34) and around 0.65 (41), respectively. Overall, it seems reasonable to conclude that latanoprost conveys the lowest risk of conjunctival hyperemia among PGAs, whereas evidence on travoprost vs bimatoprost is inconclusive.

**Table 5:** Differences between pairs of treatments in incidence of conjunctival hyperemia as determined in a network meta-analysis by Orme et al (43). Differences are expressed as odds ratios: values >1.0 indicate a greater incidence for the “row drug” vs “column drug,” and values <1.0 indicate the opposite

	Timolol	Dorzolamide	Brimonidine	Latanoprost	Travoprost	Bimatoprost
Timolol	—	∼ 1*	∼ 1*	0.56	0.18	0.11
Dorzolamide	∼ 1*	—	∼ 1*	∼ 1*	∼ 1*	0.22
Brimonidine	∼ 1*	∼ 1*	—	∼ 1*	∼ 1*	0.21
Latanoprost	1.78	∼ 1*	∼ 1*	—	0.32	0.21
Travoprost	5.55	∼ 1*	∼ 1*	3.12	—	∼ 1*
Bimatoprost	9.09	4.54	4.76	4.76	∼ 1*	—

*Odds ratios around 1 ( ∼ 1) indicate a lack of a statistically significant difference (95% confidence intervals around the odds ratio extend from below to above unity).

The review by Orme et al (43) was the only evidence of at least moderate quality about safety comparison between non-PGA compounds and indicated no relevant difference between timolol, brinzolamide, and brimonidine in respect to conjunctival hyperemia (Table 5). It also demonstrated that, in this respect, bimatoprost was considerably worse than any of these drugs, whereas latanoprost and travoprost were worse than timolol (Table 5). Data from Li et al (41) and Fung et al (33) confirmed higher incidence with travoprost vs timolol (OR 11.5) and no difference between latanoprost and brimonidine (relative risk around unity), respectively.

Fung et al (33) compared latanoprost to brimonidine in respect to a number of AEs besides hyperemia (eg, eyelid disorders, visual disturbances, keratopathy, dry eye, hypertrichosis, fatigue, headache) indicating no difference between the two. Li et al (41) indicated considerably higher odds of eye-lash changes and iris pigmentation with travoprost as compared to timolol or latanoprost. However, considering the specifics of the systematic reviews of AEs (25), in respect to these assessments quality of evidence provided by the two reviews was less than moderate: a) none of the included primary trials was specifically designed to assess safety/tolerability; b) neither review evaluated the quality of primary trials specifically in respect to AEs recording, evaluation, and reporting; c) data on most of the outcomes were available from only a few trials and prevalence of “zero event cells” was rather high.

*Combining efficacy and tolerability.* None of the five reviews (31,33,34,41,43) attempted to rank treatments based on a composite criterion combining efficacy and tolerability/safety. Data suggest that among PGAs, latanoprost has the most favorable trade-off between efficacy and tolerability. However, none of the presented reviews includes trials with preservative-free PGA formulations that have recently emerged (44-48), and this conclusion might change in the near future.

The existing evidence does not point to any relevant difference regarding efficacy and safety of non-PGA compounds – dorzolamide, brimonidine, timolol, and “other” BB. Compared to PGAs, and in addition to (at least somewhat) lower efficacy, they are limited by the fact of twice or thrice daily administration (vs once daily). It appears reasonable to consider them as alternative options when PGAs are contraindicated or not tolerated.

## Discussion

### Overall completeness and applicability of evidence

The present work addressed only a segment of pharmacological treatment of glaucoma, ie, only POAG/OHT and only mono-compound drugs, and aimed to address evidence of their relative efficacy and tolerability specifically through evaluation of systematic review/meta-analysis and not individual trials. These choices appear reasonable: a) POAG/OHT are the most prevalent conditions requiring IOP-reducing medications; b) mono-compound medications are the recommended first-line treatments; c) systematic reviews are considered the highest level of evidence based on filtered information and have been regularly published in the field. The choice of the method resulted in the fact that all currently most commonly used mono-compound drugs were embraced by the present evaluation, but some older individual drugs or drug classes (eg, older alpha2-agonists, BB, or CAI; miotics) and newer products like preservative-free formulations were not – simply due to the fact that so far they have not been subject to systematic reviews. However, these facts do not pose any major limitation to the present work. Over the years, the evaluated drugs have positioned themselves as preferable to most of the older ones and have become standards. In respect to new developments, they could be considered as “of progressively declining interest.” However, assessment of their relative efficacy and safety is of practical relevance for at least two reasons: a) full evaluation of newer or emerging treatments will take some time; b) new products, regardless of whether conveying conceptually new treatment options or “just” potential improvements to known strategies, are inevitably more expensive, particularly considering the fact that “standards” are already available in generic versions. While new options might eventually prove to be highly cost-effective, optimization of the use of pharmacological armamentarium at hand seems a reasonable effort.

### Quality of evidence

Our research question was relatively complex as it pertained to a number of individual mono-compound drugs and also to both efficacy and safety. None of the assessed reviews provided high quality evidence and five provided evidence of moderate quality. However, this judgment does not apply uniformly to all of the addressed topics. A moderate quality body of evidence was available for a) comparison between PGAs – latanoprost, bimatoprost, and travoprost in respect to efficacy and incidence of conjunctival hyperemia; b) comparison between the three PGAs and non-PGA compounds – timolol, other BB, brimonidine, and CAI (dorzolamide, or combined data for dorzolamide and brinzolamide) in respect to efficacy and conjunctival hyperemia incidence. Evidence about comparison between non-PGA compounds regarding efficacy and evidence regarding any other safety/tolerability aspect apart from conjunctival hyperemia incidence was of less than moderate quality.

### Potential biases in the overview process

All conclusions on mutual treatment comparisons in the present overview are based on evidence of moderate quality. Where this level of quality was not available, no conclusions were drawn. In this way, the conclusions are fairly protected from biases that could have been introduced by the primary trials or systematic review methodological flaws. At the overview level, we could have introduced bias by omission of one systematic review that was written in Chinese. Another source of bias could be the fact that we did not include individual studies, ie, not even those published since the last systematic review on the topic.

### Agreements and disagreements with other studies or reviews

To the best of our knowledge, this is the only overview of systematic reviews dealing with efficacy and safety of mono-compound IOP-lowering drugs in POAG/OHT.

### Conclusions

*Implications for practice.* Over the years, PGAs have emerged as preferred mono-compound treatments in POAG/OHT. The present overview indicates that among PGAs, latanoprost has the most favorable trade-off between efficacy and tolerability. Use of travoprost or bimatoprost as a first-line treatment of POAG/OHT is not very likely to result in a relevantly better efficacy, but is highly likely to result in conjunctival hyperemia, a common cause of patient-driven discontinuation of treatment. Non-PGA treatments should be considered as alternatives when PGAs are contraindicated or not tolerated. While traditionally BB, particularly timolol, have been considered as the major non-PGA treatment option, there is no clear-cut evidence that supports preference of timolol over CAI or brimonidine either regarding efficacy or regarding safety.

*Implications for research*. The medications addressed in the present overview represent the current standard choice of mono-compound IOP-lowering drugs and are likely to remain relevant for at least some time in the future. Still, certain questions about their relative efficacy and tolerability cannot be answered with certainty since the body of available evidence does not meet the criteria of at least moderate quality. With the on-going pharmacological developments in the field, these drugs are not very likely to be engaged in major primary trials in the future. However, the number of the existing primary trials – those addressed in the overviewed systematic reviews and those published over the last few years (or still on-going) – is considerable, and many of those are actually high-quality individual trials. Under such circumstances, new developments in the field of research synthesis seem a convenient and powerful tool for converting the existing primary data into evidence of relevant quality. Many of the reviews assessed in the present work suffered from serious methodological limitations, but they could all be avoided. It appears likely that adequate quality of evidence could be generated with improved assessment of quality of the primary trials, inclusion of only high-quality primary data, appropriate assessment of data combinability, sensitivity analyses, and appropriate implementation of “standard” and novel (network) data-pooling techniques.
