# Supplementary material for: Efficacy and tolerability of mono-compound topical treatments for reduction of intraocular pressure in patients with primary open angle glaucoma or ocular hypertension: an overview of reviews
Source: Croat Med J. 2014 Oct;55(5):468–80. doi: 10.3325/cmj.2014.55.468 (PMC4228301; doi:10.3325/cmj.2014.55.468)
Supplement: Supplementary Table 1 [file CroatMedJ_55_s001.pdf]

Supplementary Table 1. Records excluded after full text reading.

**Article**	**Reason for exclusion**
Hedman K et al. A pooled-data analysis of three randomized, double-masked, six-month clinical studies comparing the intraocular pressure reducing effect of latanoprost and timolol. Eur J Ophthalmol 2000; 10:95-104.	Not a systematic review, many patients with other types of glaucoma.
Hedman K et al. Pooled-data analysis of three randomized, double-masked, six-month studies comparing intraocular pressure-reducing effects of latanoprost and timolol in patients with ocular hypertension. J Glaucoma 2003;12:463-5.	The same material as above.
Qian ZG et al. Efficacy and safety of latanoprost versus travoprost for primary open-angle glaucoma and ocular hypertension: A meta-analysis. Chin J EBM 2011; 11:965-70.	Manuscript entirely in Chinese.
Beidoe G et al. Current primary open-angle glaucoma treatments and future directions. Clin Ophthalmol 2012;6:1699-707	A narrative review.
Boland MV et al. Comparative effectiveness of treatments for open-angle glaucoma: a systematic review for the U.S. Preventive Services Task Force. Ann Intern Med 2013;158:271-9	Not only RCTs, but also non-randomized and observational studies. Cited in the *Introduction*.
