# Supplementary material for: Efficacy and tolerability of mono-compound topical treatments for reduction of intraocular pressure in patients with primary open angle glaucoma or ocular hypertension: an overview of reviews
Source: Croat Med J. 2014 Oct;55(5):468–80. doi: 10.3325/cmj.2014.55.468 (PMC4228301; doi:10.3325/cmj.2014.55.468)
Supplement: Supplementary Table 2 [file CroatMedJ_55_s002.pdf]

Supplementary Table 2. Main reasons for downgrading the AMSTAR and GRADE scores.

Einarson 2000	1. The declared aim was to indirectly compare latanoprost to brimonidine. However, erroneous data pooling and meta-analysis methods were used: instead of an indirect comparison through a network (which was possible), ΔIOP vs. baseline was pooled for the two treatments across trials that compared them to a variety of other treatments, and a non-randomized comparison was performed. 2. Seemingly erroneous calculation of variance of “within-treatment” ΔIOP vs. baseline.
Zhang 2001	1. Erroneous calculation of variance of “within-treatment” ΔIOP vs. baseline and of variance of “%Δ vs. baseline” (1)*. 2. When Q test “not significant” fixed-effect pooling. However, with a small number of trials at time points with the largest treatment differences, Q-test p-value was>0.1, but we calculated I^2 values (2)[#] of 56%, 20% and 34.5%, respectively. Consequently, random-effects pooling would have been more appropriate for generation of valid estimates. 3. Handling of cross-over trials not clearly stated (possible “unit-of-analysis” issue). 4. A comprehensive overview of local and systemic safety reported in individual trials was presented. However, data pooling likely inappropriate: many “zero event cells”/”no event trials” should not be pooled as random-effects relative risk or risk difference (3).
v.d. Valk 2005	1. Erroneous data pooling and meta-analysis method (1)*: ΔIOP vs. baseline for individual treatments instead of between-treatment differences; relative treatment “positioning” based on non-randomized comparisons. 2. Erroneous calculation of variance of “within-treatment” ΔIOP vs. baseline and of variance of “%Δ vs. baseline” (1)*.
Li 2006	1. Several trials with >2 arms treated as if assessing independent pairwise comparisons (“unit-of-analysis” issue). 2. Time points that were much apart (1 week to 12 months) were pooled together (no account on trial duration) and “type” of IOP (diurnal, peak or through) not declared. This could have influenced the estimated treatment differences. 3. Some safety estimates based on only few trials with some “zero event cells”.
Denis 2007	1. Erroneous data pooling and meta-analysis method (1)*: IOP at end of treatment for individual treatments instead of between-treatment differences; relative treatment “positioning” based on non-randomized comparisons. 2. Blinding and ITT analysis by trial not addressed. 3. Inclusion of secondary OAG not stated.
Fung 2007	1. Only 4 double-blind trials, only 5 with ITT analysis. 2. Still high heterogeneity in subgroup (by trial duration) analysis. 3. A comprehensive display of adverse events, but for some high heterogeneity, for others only a few trials and “zero event cells/no event trials” (random-effects risk difference not appropriate effect measure) (3).
Aptel 2008	1. Fixed-effect pooling although Q test for some outcomes p<0.05. For others, Q test p>0.1, but with only a few trials. E.g., for two largest treatment differences we calculated I^2 values (2)[#] of 44%, respectively (random-effects might have been more appropriate). 3. One 3-arm trial – unclear handling (“unit-of-analysis” issue?). 4. Latanoprost vs. travoprost only in 1 trial. 5. No record on ITT analysis by trial. 6. Individual trial data on conjunctival hyperemia not displayed.
Cheng 2008	1. Erroneous calculation of variance of “within-treatment” ΔIOP vs. baseline and of “%Δ vs. baseline” (1)*. 2. Stated ITT analysis but, as detected through other reviews, at least 5 RCTs did not apply ITT analysis. While this can be corrected at the meta-analytical level for binary outcomes, a correction is impossible for continuous outcomes. 3. Efficacy treatment difference inconsistent for morning and diurnal IOP, as well as for “% achieving target”. 4. Handling of cross-over trials not stated (possible “unit-of-analysis” issue). 5. Comprehensive presentation of adverse effects, but some only on few trials with “zero event cells” (random-effects risk difference not an appropriate effect measure) (3).
Hodge 2008	1. 2/4 trials comparing efficacy of latanoprost to brimonidine and 3/3 comparing it to brinzolamide - low quality (Jadad score 2). 2. Only 1 study for each comparison was based on ITT analysis. 3. All trials for latanoprost vs. dorzolamide on diurnal IOP, latanoprost vs. brimonidine on diurnal and morning mixed together (might have affected the estimates). 4. Safety outcomes assessed on only a few trials. Individual trial data not displayed, but likely some “zero event cells”/”no event trials” (risk difference not appropriate effect measure) (3).
Loon 2008	1. Erroneous data pooling: efficacy subgroup analysis by trial size using fixed-effect despite very high I^2 concluded treatment difference in large trials. Re-calculated by random-effects – no difference I. 2. Erroneous weighing in subgroup analysis by trial duration (relative weight distributed as if there were 16 trials). 3. Unclear meta-regression procedure (could not be replicated)H.4. Safety outcomes assessed on trials with high prevalence of “zero event cells/trials” (random-effects relative risk not appropriate effect measure) (3).
Cheng 2009a	1. Erroneous calculation of variance of “within-treatment” ΔIOP vs. baseline (1)*. 2. Stated ITT, but 11/17 included RCTs did not apply ITT analysis. While this can be “corrected” at the meta-analytical level for binary outcomes, a correction is impossible for continuous outcomes. 3. Handling of cross-over trials not stated (possible “unit-of-analysis” issue). 4. No sensitivity analysis in respect to ITT and blinding. 5. Inconsistent results for diurnal and afternoon IOP reduction. 5. A comprehensive display of adverse events, but some assessed on only a few trials. Unknown prevalence of “zero event cells”/”zero event trials” (random-effects relative risk not appropriate effect measure) (3).

Cheng 2009b	1. Erroneous data pooling and meta-analysis method (1)*: ΔIOP vs. baseline for individual treatments instead of between-treatment differences; relative treatment "positioning" based on non-randomized comparisons. 2. Erroneous calculation of variance of "within-treatment" ΔIOP vs. baseline and of variance of "%Δ vs. baseline" (1)*.
Ejawo 2009	1. Aimed to include only RCTs, but 4/16 trials were non-randomized. 2. The 5 3-arm trials handled as if independently assessing pairwise comparisons - "unit-of-analysis" issue. 3. No reference to individual study blinding, randomization and ITT analysis. 4. Individual study data on conjunctival hyperemia not displayed.
Hornubia 2009	1. One 3-arm trial treated as if assessing independent pairwise comparisons – "unit-of-analysis" issue. 2. Unclear handling of cross-over trials (further "unit-of-analysis problem"?). 3. ITT approach and blinding by study not addressed.
v.d.Valk 2009	1. A network ("multiple-treatment") meta-analysis based on previously identified (van der Valk et al. 2005) RCTs – likely erroneous (as in the previous publication) calculation of variance of "%Δ vs. baseline" .2. No up-dated trial search. 3. Ranking based on individual treatment difference vs. timolol, but a limited number of direct comparisons for many treatments in the network. 4. Traditional meta-analysis not performed. 5. ITT analysis and blinding by trial not addressed.
Orme 2010	1. A network ("multiple-treatment") meta-analysis using latanoprost as a reference, but with a limited number of direct comparisons for many treatments in the network. 2. ITT analysis and blinding by trial not addressed. 3. Classical meta-analysis not performed.

*Reported in a previous assessment (1);

$I^2 = 100\% \times \frac{Q - df}{Q}$

I In STATA (metan), CMA and R (metafor), random-effects meta-analysis by trial size subgroups based on displayed data.

H Using displayed data on trial size (>100 or <100; or continuous), duration (<6 months or >6 months; or continuous) and allocation concealment (yes/no), could not replicate the reported meta-regression results (SAS proc mixed; metareg in STATA; metafor in R).

IOP= intraocular pressure; ITT= intention-to-treat.

1. Li T, Dickersin K. Citation of previous meta-analyses on the same topic. A clue to perpetuation of incorrect methods? Ophthalmol 2013; 120:1113-9.

2. Higgins JPT, Thomson SG, Deeks JJ, Altman DA. Measuring inconsistency in meta-analyses. BMJ 2003; 328:557-60.

3. Higgins JPT, Deeks JJ, Altman DG. Special topics in statistics. In: Cochrane Handbook for Systematic Reviews of Interventions, version 5.1.0, Higgins JPT, Green S (eds) [Internet]. The Cochrane Collaboration; 2011 [updated March 2011]. Available from www.cochrane-handbook.org.
